# Identification and Genome Characterization of Novel Feline Parvovirus Strains Isolated in Shanghai, China

**DOI:** 10.3390/cimb45040236

**Published:** 2023-04-20

**Authors:** Chengqian Liu, Fusheng Si, Hong Li, Jun Gao, Fengping Sun, Huili Liu, Jianzhong Yi

**Affiliations:** Institute of Animal Science and Veterinary Medicine, Shanghai Academy of Agricultural Sciences, Shanghai 201106, China; liuchengqian@saas.sh.cn (C.L.); mr.fusheng@163.com (F.S.); lihong20061029@163.com (H.L.); gaojun@saas.sh.cn (J.G.); sfp_hot@163.com (F.S.)

**Keywords:** feline panleukopenia virus (FPV), genome characterization, structural proteins, phylogenetic analysis, genetic evolution

## Abstract

Feline panleukopenia virus (FPV) is the causative agent of hemorrhagic gastroenteritis in feline animals. FPV has been evolving over time, and there have been several different strains of the virus identified. Some of these strains may be more virulent or more resistant to current vaccines than others, which highlights the importance of ongoing research and monitoring of FPV evolution. For FPV genetic evolution analysis, many studies focus on the main capsid protein (VP2), but limited information is available on the nonstructural gene NS1 and structural gene VP1. In the present study, we firstly isolated two novel FPV strains circulating in Shanghai, China, and performed full-length genome sequencing for the desired strains. Subsequently, we focused on analyzing the NS1, VP1 gene, and the encoding protein, and conducted a comparative analysis among the worldwide circulating FPV and Canine parvovirus Type 2 (CPV-2) strains, which included the strains isolated in this study. We found that the 2 structural viral proteins, VP1 and VP2, are splice variants, and VP1 has a 143 amino-acid-long N-terminal compared to VP2. Furthermore, phylogenetic analysis showed that divergent evolution between FPV and CPV-2 virus strains were clustered mostly by country and year of detection. In addition, much more continuous antigenic type changes happened in the process of CPV-2 circulating and evolution compared to FPV. These results stress the importance of the continuous study of viral evolution and provide a comprehensive perspective of the association between viral epidemiology and genetic evolution.

## 1. Introduction

The feline panleukopenia virus (FPV) is classified as a parvovirus based on its infectious and non-enveloped characteristics as well as its single-stranded DNA structure. It is capable of infecting various animals, including domestic cats, raccoons, and minks. The virus typically induces acute gastroenteritis and leukopenia, resulting in clinical signs such as acute diarrhea. FPV poses a significant threat to young animals as it has a high mortality and morbidity rate in this population [1,2,3]. The genome of FPV is approximately 5.1 kb in length and has two open reading frames (ORFs), ORF1 and ORF2. ORF1 encodes two non-structural proteins, nonstructural protein 1 (NS1) and nonstructural protein 2 (NS2). NS1 is a polyprotein-encoding, ORI binding domain, helicase, and transactivation functional domains; the NS2 protein is generated from the fusion of a 260-nt left-hand fragment and a 238-nt right-hand fragment of the NS1 open reading frame [4,5]. The ORF2 region encodes two capsid proteins, VP1 and VP2. These proteins are splice variants and are nearly identical in sequence, except for a unique 143 amino-acid N-terminal stretch in VP1 [6,7]. VP2 is the predominant capsid protein, constituting about 90% of the entire viral capsid, and is the main target for neutralizing antibodies against parvovirus, thereby determining host range [8]. Specific amino acids in the VP2 protein are crucial for defining host range differences between feline panleukopenia virus (FPV) and canine parvovirus type 2 (CPV-2). These amino acid positions include 80 (Lys to Arg), 93 (Lys to Asn), 103 (Val to Ala), 232 (Val to Ile), 323 (Asp to Asn), 564 (Asn to Ser), and 568 (Ala to Gly) [9].

The VP2 protein determines the antigenicity of FPV or CPV-2. Mutations affecting VP2 are mainly responsible for the evolution of different antigenic variants of CPV-2 and FPV [5]; therefore, sequencing and phylogenetic analysis of the full VP2 gene and full-length FPV genome will provide information on the prevailing antigenic types of FPV. Despite the potential occurrence of consecutive genetic evolution, there is a paucity of global data on the genomic analysis of feline panleukopenia virus (FPV) and canine parvovirus type 2 (CPV-2) strains. Additionally, there has been no comprehensive genetic analysis conducted on FPV isolates in China [10,11,12].

In the current study, we conducted a comprehensive genetic analysis of feline panleukopenia virus (FPV) strains isolated in Shanghai, as well as recent FPV isolates circulating throughout China. Furthermore, we thoroughly characterized two FPV strains that were isolated from domestic cats exhibiting gastroenteritis. Our genome-wide analysis revealed that these FPV isolates can be differentiated from other currently circulating FPV isolates in China, based on variations in several nucleotides and unique amino acid coding in the NS1 and VP2 genes. These findings provide new insights into the correlation between FPV epidemiology and genetic evolution.

## 2. Materials and Methods

### 2.1. Fecal Samples Collection

In this study, 20 fecal specimens from cats with clinical signs of suspected parvovirus infection were collected in 2020 from 2 veterinary clinics in Minhang districts of Shanghai city, China. All samples were procured in accordance with the Chinese legislation governing animal welfare and the guidelines stipulated by the Chinese policies and practices on veterinary medicine, with the explicit consent of the owners.

### 2.2. Sample Treatment and Amplification of FPV

Collection tubes were used to collect samples, which were then subjected to TRFIA (time-resolved fluoro-immunoassay) analysis to ascertain the presence of FPV [13,14]. To this end, fecal samples were suspended in PBS, and the resulting supernatants were added to the test strips at ambient temperature. A Virus DNA Kit was utilized to extract total viral DNA from the fecal samples as per the manufacturer’s instructions. This extracted DNA was subsequently employed for FPV detection via polymerase chain reaction (PCR) utilizing a primer pair (P1: 5′-GAATCTGCTACTCAGCCAAC-3′ and P2: 5′- GTGCACTATACCACCACCTCAGC-3′). The polymerase chain reaction (PCR) was performed in a reaction volume of 25 μL consisting of 2.0 μL of template, 1.0 μL each of P1 and P2 primers, 2× Ex Taq mix (TaKaRa Biotechnology Co., Ltd., Shiga, Japan), and adequate ddH_2_O to increase the final volume to 25 μL. The amplification program was as follows: initial denaturation at 95 °C for 5 min followed by 35 cycles of denaturation at 95 °C for 50 s, annealing at 55 °C for 30 s, extension at 72 °C for 30 s, and a final extension at 72 °C for 5 min. The amplified products were visualized with electrophoresis of 10 μL aliquots in 1.0% agarose gels in 1× TAE buffer (40 mM Tris-acetate (pH 8.0), 1 mM EDTA).

### 2.3. Cell Cultivation and Virus Isolation

Feline kidney (F81) cells were procured from Procell Life Science & Technology Co., Ltd. (Wuhan, China) and maintained in Dulbecco’s modified Eagle’s medium (DMEM; Gibco, Invitrogen, Carlsbad, CA, USA), supplemented with 10% fetal bovine serum (FBS; Gibco, Invitrogen), penicillin (100 U/mL), and streptomycin (100 μg/mL) at 37 °C in a humidified atmosphere containing 5% CO_2_. For virus isolation, we added 5 times the volume of PBS into the fecal samples from cats that tested positive via TRFIA and PCR and mixed them well before centrifuging and filtering the supernatant with 0.22 μM filters (Millipore, Burlingto, MA, USA). The supernatant of the filtered sample was inoculated into F81 cells according to 10% of the total volume of the cell culture medium through a synchronous inoculation method. The cells were incubated under controlled conditions at a temperature of 37 °C in a CO_2_-enriched incubator. Upon completion of four consecutive passages, the cytopathic effect of the cells reached 80%. The control group cells exhibited uniform and complete boundaries, indicative of their stable morphology. The viruses were isolated after undergoing three rounds of plaque purification through primary F81 cell culture. The culture medium was harvested once a typical cytopathic effect (CPE) was observed in 80% of the cells. The medium was then frozen and stored at −80 °C for further processing.

### 2.4. Indirect Immunofluorescence Assay (IFA)

F81 cells were inoculated into 96-well plates and subsequently exposed to the isolated FPV strains followed by an incubation period of 36 h at a temperature of 37 °C under 5% CO_2_. The isolated viruses FPV-SH2001 and FPV-SH2002 were, respectively, inoculated into cells at the same time. After 72 h, the medium was discarded, fixed with 75% cold ethanol for 30 min, washed with PBST buffer 3 times, and incubated with mouse anti-feline parvovirus monoclonal antibody (provided by Biocare Diagnostics Ltd., Zhuhai, China) as the primary antibody for 2 h and FITC-conjected Goat anti-mouse IgG (provided by Amyjet scientific Co., Ltd., Wuhan, China) as the secondary antibody for 1 h. After three washings with PBS, fluorescence was observed using a fluorescence microscope (Axio Scope A1; Carl Zeiss, Jena, Germany). Negative controls were also prepared following an identical protocol.

### 2.5. Genome Sequencing of Isolated FPV Viruses

The fourth generation of FPV-SH2001 and FPV-SH2002 culture medium was used to extract the virus DNA according to the instructions of the genomic DNA extraction kit (Takara, Beijing, China). The DNA was used as the template for PCR amplification. The PCR system assays used KOD high fidelity DNA polymerase (Toyobo, Osaka, Japan), the reaction volume was 50 μL: DNA template 3 μL 10 × buffer (Mg^2+^ free) 5 μL, MgSO_4_ 4 μL, dNTP (2 mM) 5 μL, forward and reverse primers (10 mM) 1.5 μL each, KOD plus enzyme 2 μL, ddH_2_O 28 μL; mixed on ice. The PCR cycle parameters were 94 °C for 5 min, 94 °C for 45 s, 55 °C for 30 s, 68 °C for 180 s, 35 cycles, and finally 68 °C for 5 min. The amplified products of 6 μL were identified using 1% agarose gel electrophoresis. The PCR products were sent to BioSune Biotechnology (Shanghai, China) Co., Ltd. for sequencing, and the verified sequences of FPV-SH2001 and FPV-SH2002 were submitted to the NCBI GenBank database and were assigned the accession numbers MW650831 and MW659466.

### 2.6. Phylogenetic Analysis

In the first stage of our study, we utilized the BLAST suite, specifically blastn and blastx algorithms, to detect viral sequences by matching them against annotated genomes present in the Genebank database. After identifying the most reliable hits from the blastn search, we selected the complete genomes of carnivorous and feline parvoviruses to be subjected to subsequent analyses. The Clustal X software (version 2.0) was employed to align these genomes, and a phylogenetic tree was subsequently constructed using the Maximum Likelihood method with 1000 bootstrap replicates. All phylogenetic analyses and tree editions were conducted using MEGA-X software (version 10.1.8) [15].

### 2.7. The Recombination Event Detection

Recombination analysis was performed on FPV isolates using the Recombination Detection Program (version 4.95). First, the Clustal W algorithm was employed to align the nucleotide sequences of FPV isolates. The resulting alignment was analyzed using RDP v4.95 to identify potential recombination events, with the following parameters applied: Bonferroni correction for multiple testing, a minimum length of 100 bp for potential recombinants, and all available detection methods (RDP, GENECONV, Bootscan, Maxchi, Chimaera, and SiScan). The highest-scoring recombination events were manually checked from the RDP output. The RDP v4.95 program was then used to determine the recombination breakpoints and the mosaic structure of the putative recombinant sequences. Finally, the RDP screenshots displayed the 99% and 95% confidence intervals for predicting breakpoints.

## 3. Results

### 3.1. Detection and Identification of Novel FPV Strains

Fecal samples were suspended in PBS and the resulting supernatants were filtered and added to TRFIA test strips at room temperature. The left panels of the figure depict the outcome of the TRFIA test strip, wherein the control band appears at the top and the testing band at the bottom. Fecal samples obtained from sick cats displayed positive results, as indicated by the lower band (Figure S1). Additionally, PCR screening was performed on the samples, with the PCR-positive samples exhibiting a 559 base pair band, amplified using previously described general test primers (Figure S1).

### 3.2. Virus Isolation, Proliferation, and IFA Assay

**Figure 1:**
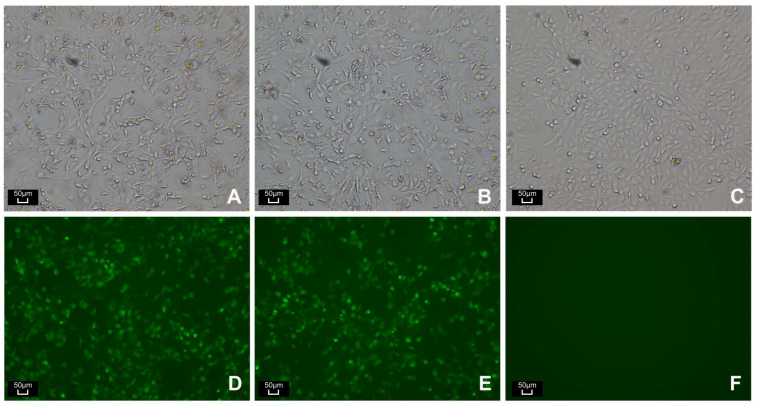
The cytopathic effect caused by FPV isolate and the indirect immunofluorescence assay result on the infected cells. (**A**) Typical cytopathic morphological changes in FPV-SH2001-infected F81 cells. (**B**) Typical cytopathic morphological changes in FPV-SH2002-infected F81 cells. (**C**) Mock infection of the normal F81 cells. (**D**) The immuno-fluorescence assay of the FPV-SH2001-infected cells. (**E**) The immuno-fluorescence assay of the FPV-SH2002-infected cells. (**F**) The normal F81 control cells.

A total of 5 times volume of PBS was used to dilute the cat fecal samples, which were tested positive via PCR, mixed well, the supernatant was centrifuged and filtered with 0.22 μM filters. The supernatant of the filtered samples was inoculated into F81 cells as 10% of the total volume of the cell culture medium via a synchronous inoculation method. The cells were cultured in a 37 °C incubator containing 5% CO_2_. After four passages of blind passage, about as many as 80% of the inoculated cells showed cytopathy state; however, the control cells showed complete boundary and uniform morphology (Figure 1). They are named FPV-SH2001 and FPV-SH2002, respectively. IFA fluorescence was observed through a fluorescence inverted fluorescence microscope; green fluorescence could be observed in the infected cells, indicating that the isolated strain could react specifically with FPV antibody (Figure 1).

### 3.3. Genomic Sequencing of Isolated FPV Strains

**Figure 2:**
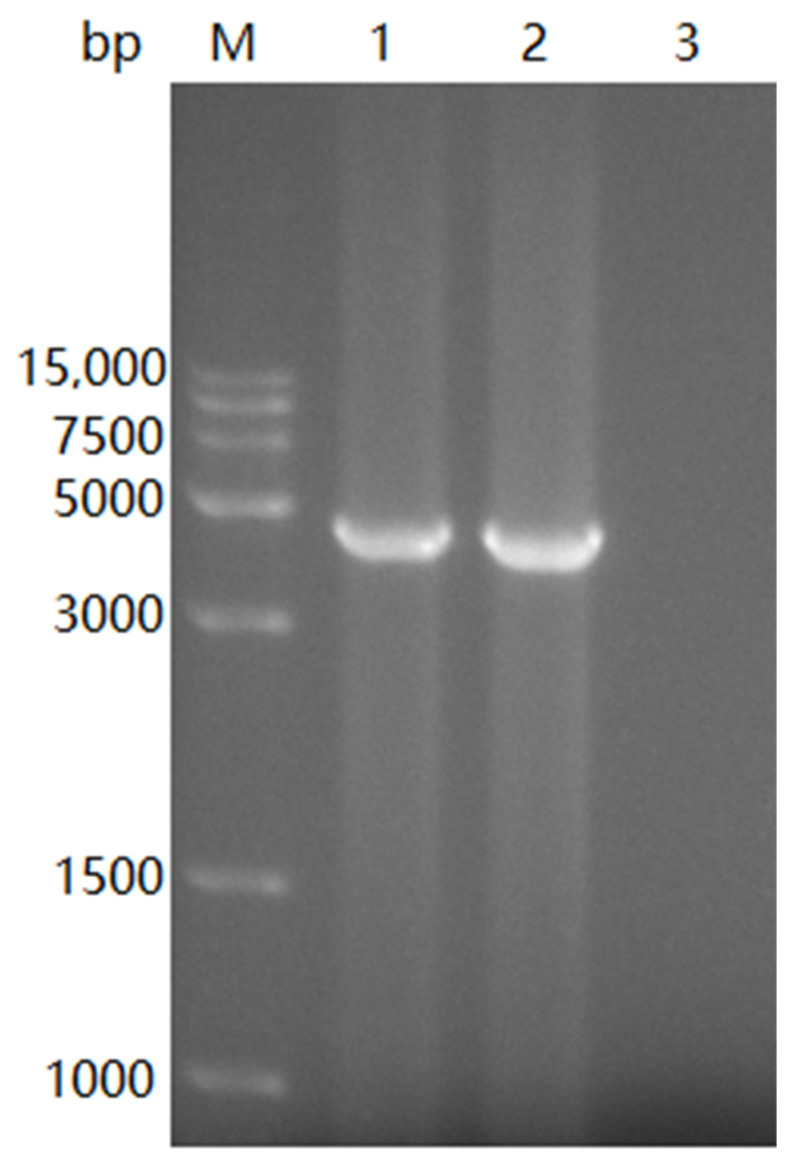
The full genome amplification of the isolated FPV. Full-length sequences of FPV Shanghai strains were obtained from the infected F81 cells through PCR. The near-full genome amplification band are shown at about 4811 bp. Lane M: DNA Marker 15000; Lane 1: FPV-SH2001; Lane 2: FPV-SH2002; and Lane 3: negative control.

The near-full genome amplification bands are shown at about 4811 bp (Figure 2). The molecular characteristics and homology of nucleotide sequences of the nearly entire viral genome were assembled, and the deduced amino acids were analyzed with the MegAlign module of DNASTAR software (version 7.0).

### 3.4. Phylogenetic and Comparative Analysis of NS1 Gene of the Isolated FPV Strains and Other Worldwide FPV Strains

The nonstructural proteins NS1 and NS2 are essential for viral replication, DNA packaging, cytotoxicity, and pathogenicity [16,17]. Most studies have focused on the VP2 gene of the CPV-2 and FPV strains [18]; however, there has been limited attention paid to the nonstructural genes of NS1 and NS2 [19,20]. To better elucidate the molecular features of NS1 and its role in the evolution of FPV, we conducted a phylogenetic study to characterize NS1 sequences of the isolated FPV strains and compare them with available NS1 sequences of FPV strains worldwide. Our study found that polymorphic amino acid sites laid in D23N, Q247H, I443V, E545V, Q595H, and L596V. In comparative analysis of FPV NS1 proteins, these varied residues putatively lie in the interface among the ORI binding domain, helicase, and transactivation functional domains (Table 1). When comparative analysis of FPV NS1 proteins with CPV-2 strains was performed, the differences between the FPV and CPV-2 strains were found at amino acid residues 19, 23, 60, 231, 247, 248, 438, 443, 544, 545, 574, 588, 596, 630, and 665 (Table 1). Polymorphic amino acid sites of FPV were laid in D23N, Q247H, I443V, E545V, and L596V. In contrast, CPV-2 strains showed polymorphic amino acid sites at R19K, V60I, F544Y, V545E, M574I, I588N, P630L and G665D. The only exception was amino acid site 248 in which T is in the FPV strains and I is in the CPV-2 strains. These convergent or divergent amino-acid changes between FPV and CPV-2 could contribute to further elucidate the NS1 protein structure by clarifying the potential role of these residues [21,22].

**Figure 3:**
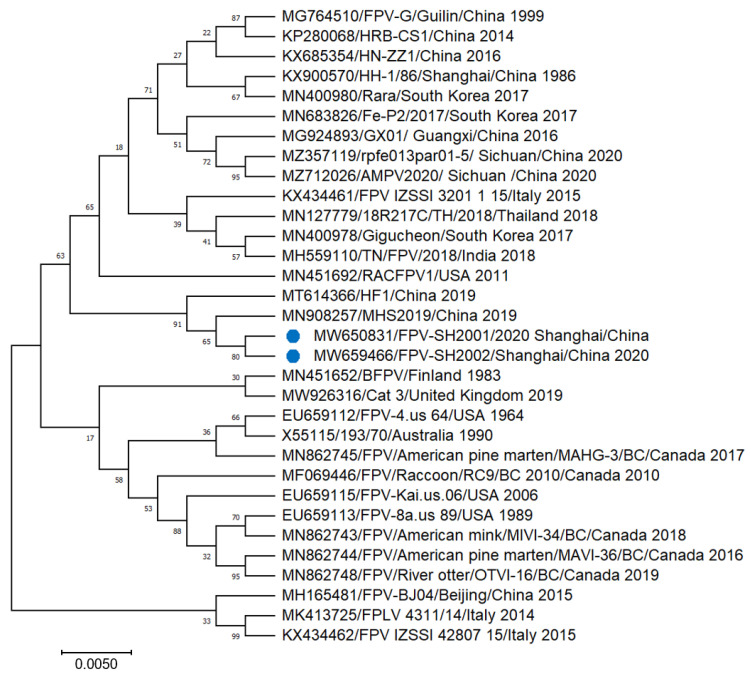
Phylogenetic analyses of NS1 gene nucleotide sequences from the isolated FPV strains in this study. The phylogenetic tree was inferred using the Maximum Likelihood method with 1000 bootstrap replicates, using the MEGA-X software (version 10.1.8). Blue circles designate the FPV sequences identified from the present study. The remaining ones are the reference sequences.

The NS1 gene phylogenetic tree demonstrated that FPV-SH2001 and FPV-SH2002 are in same phylogenetic branch. The analyzed sequences clustered mainly according to viral lineage and also relative to the geographical area and the year of sample collected, suggesting divergent evolution among FPV strains for the NS1 gene (Figure 3). The FPV-NS1 protein’s phylogenetic tree displays two distinct branches that possess strong confidence values, indicating a clear segregation of evolutionary lineages (Figure S2). The present isolated MW650831 FPV-SH2001 and MW659466 FPV-SH2002 clustered closely related to China-MT614366 and China-MN908257, which suggests that these branches may have arisen from a common ancestor and subsequently diverged from each other (Figure 3 and Figure S2). The analyzed NS1 gene and encoding protein demonstrated almost the same phylogenetic tree, suggesting limited non-synonymous mutation occurred during the process of FPV evolution [23,24].

### 3.5. Phylogenetic and Comparative Analysis of VP2 Gene of the Isolated FPV Strains and Other Worldwide FPV Strains

ORF2 encodes two capsid proteins, VP1 and VP2. The 2 structural viral proteins are splice variants and are identical in sequence except for a 143 amino acid (aa)-long N-terminal stretch that is unique to VP1 [25]. The VP2 is the major capsid protein that comprises about 90% of the entire viral capsid. VP2 proteins are exposed on the surface of the capsid and determine the antigenicity of CPV-2 and FPV, which contains neutralizing epitopes [26]. In the present study, polymorphic amino acid sites were laid in A5G, K93N, A103V, V232I, Q370R, A411D/E, D426E/N, A440T, and A568G in comparative analysis of FPV VP2 proteins. In comparative analysis of VP1 proteins, both the FPV and CPV-2 strains, differences between FPV and CPV-2 strains were found at aa residues 20, 24, 31, 140, 172, 247, 254, 258, 260, 270, 399, 434, 464, 467, 472, 490, 491, 537, 593, 731, and 735 (Table 2). Three polymorphic amino acid sites of FPV were laid in the VP1 N-terminal at L24I, G31C. Only one polymorphic amino acid site laid in VP2 at A258S, which indicates that the VP2 gene in CPV-2 possesses a much higher evolution rate compared to FPV, which may be the result of its different host range. Moreover, specific aa residues for FPV and CPV-2, respectively, laid in 467A, 472D, 490D, 731N, and 735A (FPV) and 467G, 472Y, 490N, 731S, and 735G (CPV-2). Some polymorphic amino acid sites were relative to neutralization antigenic epitopes of different FPV and CPV-2 strains.

**Figure 4:**
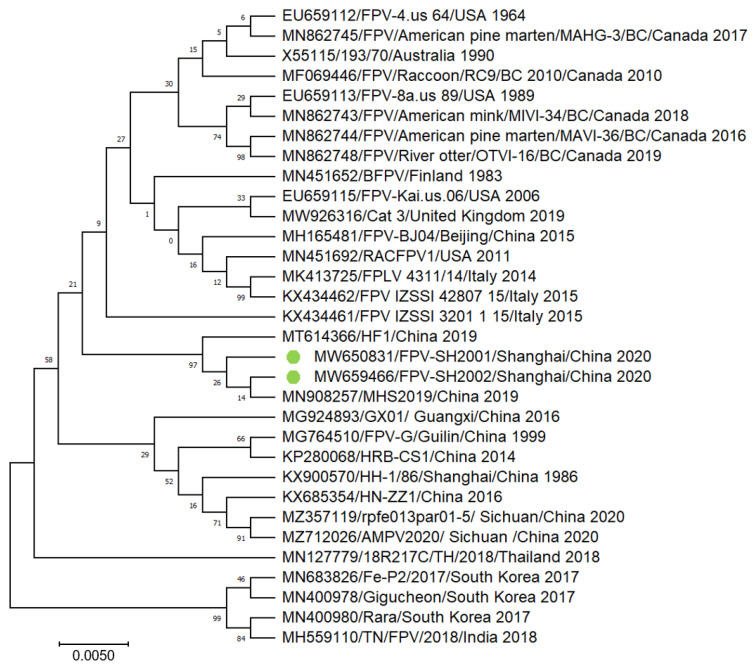
Phylogenetic analyses of VP2 gene nucleotide sequences from the isolated FPV strains in this study. The phylogenetic tree was inferred using the Maximum Likelihood method with 1000 bootstrap replicates, using the MEGA-X software (version 10.1.8). Green circles designate the FPV sequences identified from the present study. The remaining ones are the reference sequences.

Based on the results of the phylogenetic analysis of the VP2 gene of viruses MW650831 FPV-SH2001 and MW659466 FPV-SH2002, it was observed that these viruses were in close proximity to the China-MT614366 and China-MN908257 viruses (Figure 4). It was further observed that all the analyzed FPV strains were grouped in one clade. These findings suggest that the FPV strains possess a high degree of similarity and are closely related to other FPV strains found in the region.

### 3.6. Phylogenetic Analysis of Whole Genome Sequences of the Isolated FPV Strains and Other Worldwide FPV Strains

**Figure 5:**
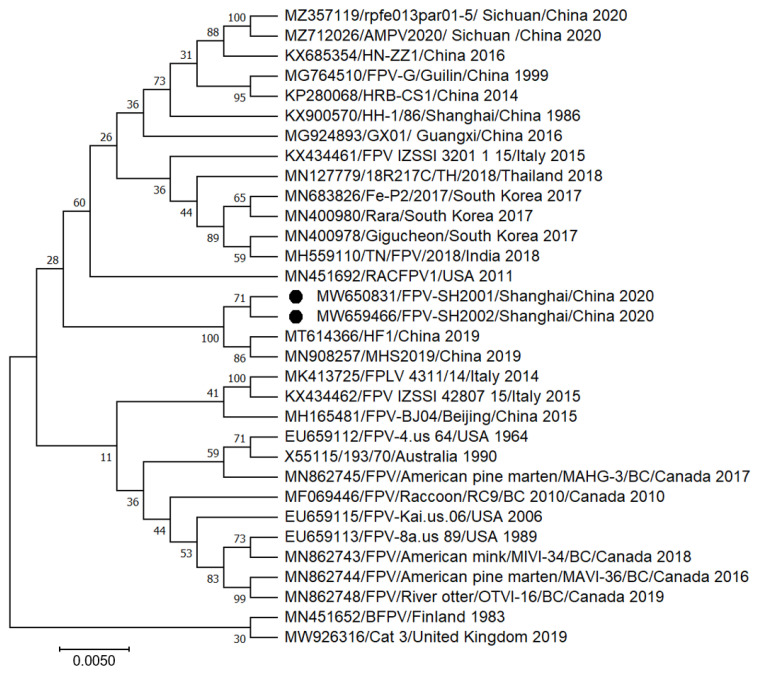
Phylogenetic analyses of whole genome sequences from the isolated FPV strains in this study. The phylogenetic tree was inferred using the Maximum Likelihood method with 1000 bootstrap replicates, using the MEGA-X software (version 10.1.8). Black circles designate the FPV sequences identified from the present study. The remaining ones are the reference sequences.

As per the outcomes of the phylogenetic analysis carried out on the complete genome sequences of MW650831 FPV-SH2001 and MW659466 FPV-SH2002, it has been established that these strains are thoroughly interconnected with China-MT614366 and China-MN908257, and all the FPV strains scrutinized during the study belong to a single clade (Figure 5). The data revealed by this study confirm that the isolated strains share a close relationship with other FPV strains from China, which could have important implications for the spread and evolution of FPV.

## 4. Discussion

Feline panleukopenia (FPV), which is caused by the parvovirus, is a highly contagious viral disease among cat animals. The responsible pathogen is an unenveloped DNA virus with a single strand of a small size. It has the ability to infect domestic cats as well as other species in the Felidae family. Additionally, it can also infect animals belonging to the families Procyonidae, Mus-telidae, and Viverridae, including foxes, minks, and ring-tailed cats [1,27]. The virus causes a disease in cats characterized by enteritis accompanied by intestinal villus degeneration and a severe decrease in circulating white blood cell count, which is also known as feline panleukopenia. This infection is highly contagious and closely related to high morbidity and mortality among cat animals [1,28,29]. Therefore, timely follow-up, isolation, and monitoring of genetic evolution of FPV field strains are of great importance for further development of prevention and control strategies [30,31].

In the current study, based on the analysis of NS1 and VP2 gene sequences and the evolution of the full genome, the genetic evolutionary distance and relationship of the two FPV isolates are consistent. In addition, isolates FPV-SH2001 (MW650831) and FPV-SH2002 (MW659466) are closely related to the Chinese strains MHS2019 (MN908257) and HF1 (MT614366). Furthermore, the NS1, VP1 gene, and the corresponding encoding protein were determined from the desired strains. We found that the 2 structural viral proteins, VP1 and VP2, are splice variants, and VP1 has a 143 amino-acid-long N-terminal compared to VP2. Furthermore, we found that the polymorphic amino acid sites were laid in D23N, Q247H, I443V, E545V, and L596V. In comparative analysis of FPV NS1 proteins, varied residues putatively laid in the interface among the ORI binding domain, helicase, and transactivation functional domains. According to current knowledge, the mechanism underlying the species specificity of CPV and FPV appears to be related to the differences in their capsid proteins, which differ by only eight to ten amino acid residues. CPV-2 evolved from FPV by altering the species-specific binding of the viral capsid to the host receptor, i.e., the transferrin receptor (TfR). Therefore, it can be concluded that specific amino acid residues in the VP2 protein of CPV-2 and FPV are related to the host tropism. In addition, much more continuous antigenic type changes occurred in the process of CPV-2 circulating and evolution than FPV. These results stress the importance of the continuous study of viral evolution and provide a comprehensive perspective of the association between viral epidemiology and genetic evolution.

We then constructed phylogenetic analyses based on NS1 and VP2 genome sequences and amino acid sequences, respectively, to determine the relatedness and heterogeneity between the two isolates and other reference FPV strains. A phylogenetic tree based on NS1 genome reconstruction showed that the two isolate strains were grouped into one separate cluster together with other two Shanghai isolates, MHS2019 (MN908257) and HF1 (MT614366). In addition, we found that the two Shanghai FPV isolates were grouped in a novel cluster separated from FPV from other countries, regardless of whether the NS1 or VP2 gene is used for phylogenetic analysis (Figure 3 and Figure 4). Based on the full-length NS1 or VP2 genomes analysis at the nt level, we found that the genetic similarity between the two Shanghai isolates ranges from 98.3 to 99.7%. Further analysis showed that although the two Shanghai isolates belonged to the same cluster; they were located in different branches (Figure 4), indicating that the FPV of Shanghai isolates were two different individuals but closely related to each other according to the genetic relationship, and this close relationship between the FPV-SH2001 and FPV-SH2002 isolates was also confirmed by the subsequent genetic analyses. In addition, based on the amino acid sequence alignment of NS1 and VP1, we confirmed that the two Shanghai FPV isolates possessed high genetic similarity with FPV-BJ04/2015 and FPV/GD (12/09/YGP), respectively. This indicated that even though the two isolates underwent genetic variation at a few amino acid loci, they possess a close genetic evolutionary relationship and probably originate from the same ancestral branch.

Recombination has long been recognized as an important evolutionary mechanism in many virus families, having the potential to adapt to harsh environments and obtain batter survival opportunities [32,33]. Previous studies have suggested the occurrence of recombination between FPV and CPV-2 [25]. In our study, no recombination signal was found for either FPV-SH2001 or FPV-SH2002 with other referenced FPV strains (Tables S1 and S2), indicating that the isolated FPVs are not a recombination of other parvoviruses. However, further study is still required to determine whether FPV is genetically related to other intrageneric viruses with similar ancestors and to address the exact phylogenetic relationships between various feline parvovirus species and strains.

## 5. Conclusions

This study aimed to analyze the NS1 and VP1 genes, as well as their encoding proteins, and to report on the comparative differences among worldwide circulating strains of FPV and CPV-2. We firstly isolated two FPV strains circulating in Shanghai, China; a full-length genome amplification was performed and sequenced. Phylogenetic analysis showed divergent evolution between FPV and CPV-2. Virus strains were clustered mostly by country and year of detection. Much more continuous antigenic type changes occurred in the process of CPV-2 circulating and evolution compared to FPV. Our results help understand the functional impact of these genomic changes on the biology, immunology, and pathogenesis of FPV.

## Figures and Tables

**Table 1:** Amino-acid variation of NS1 protein in the isolated strains.

Isolate			Mutated Loci of Amino Acid														
Names	GenBank ID	Subtypes	19	23	60	231	247	248	438	443	544	545	574	588	596	630	665
FPV-SH2001	MW650831	FPV	K	D	I	S	H	T	K	V	Y	E	I	S	L	L	D
FPV-SH2002	MW659466	FPV	K	D	I	S	H	T	K	V	Y	E	I	S	L	L	D
HRB-CS1	KP280068	FPV	.	N	.	G	Q	.	E	I	.	.	.	.	V	.	.
HN-ZZ1	KX685354	FPV	.	N	.	.	Q	.	K	I	.	.	.	.	V	.	.
HH-1/86	KX900570	FPV	.	N	.	.	Q	.	K	I	.	.	.	.	V	.	.
HF1	MT614366	FPV	.	.	.	.	.	.	E	I	.	.	.	.	.	.	.
CPV-LZ1	JQ268283	CPV-2a	.	N	.	.	Q	I	.	I	.	.	.	N	V	.	.
CPV-JS2	KF676668	CPV-2a	.	N	.	.	Q	I	.	I	F	V	.	I	V	.	.
CPV/CN/ya1/2017	MG583676	CPV-2a	R	N	.	.	Q	I	.	I	F	V	.	I	V	.	.
9985-46	LC270892	CPV-2b	R	N	.	.	Q	I	.	I	.	.	M	I	V	.	G
CPV-SH1515	MG013488	CPV-2c	.	N	V	.	Q	I	.	I	F	V	.	I	V	P	.
CPV-AHhf1	MT010564	CPV-2c	.	N	V	.	Q	I	.	I	F	V	.	I	V	P	.
Taiwan/2018	MN832850	CPV-2c	.	N	V	.	Q	I	.	I	F	V	.	I	V	P	.

Note: The dots (.) represent amino acid residues that are identical to those found in the sequences of FPV-SH2001 and FPV-SH2002.

**Table 2:** Amino-acid variation of the VP1 protein in isolated strain.

Isolate			Mutated Loci of Amino Acid																				
Names	GenBank ID	Subtypes	20	24	31	140	172	247	254	258	260	270	399	434	464	467	472	490	491	537	593	731	735
FPV-SH2001	MW650831	FPV	E	I	C	K	A	K	M	S	K	V	V	F	S	A	D	D	Y	Q	N	N	A
FPV-SH2002	MW659466	FPV	E	I	C	K	A	K	M	S	K	V	V	F	S	A	D	D	Y	Q	N	N	A
HRB-CS1	KP280068	FPV	.	L	.	K	.	.	.	A	.	.	.	.	.	.	.	.	.	.	.	.	.
HN-ZZ1	KX685354	FPV	.	L	.	.	.	.	.	A	.	.	.	.	.	.	.	.	.	.	.	.	.
HH-1/86	KX900570	FPV	.	L	.	.	.	.	.	A	.	.	.	.	.	.	.	.	.	.	.	.	.
HF1	MT614366	FPV	.	.	G	.	.	.	.	.	.	.	.	.	.	.	.	.	.	.	.	.	.
CPV-LZ1	JQ268283	CPV-2a	.	L	.	.	.	R	L	A	N	A	I	.	A	G	Y	N	I	.	.	S	G
CPV-JS2	KF676668	CPV-2a	.	L	.	R	.	R	L	A	N	A	I	Y	A	G	Y	N	I	.	.	S	G
CPV/CN/ya1/2017	MG583676	CPV-2a	.	L	.	R	.	R	L	A	N	A	I	Y	A	G	Y	N	I	.	.	S	G
9985-46	LC270892	CPV-2b	K	L	.	.	.	R	L	A	N	A	I	.	.	G	Y	N	.	.	D	S	G
CPV-SH1515	MG013488	CPV-2c	.	L	Y	.	G	R	L	A	N	A	I	Y	A	G	Y	N	I	R	E	S	G
CPV-AHhf1	MT010564	CPV-2c	.	L	Y	.	G	R	L	A	N	A	I	Y	A	G	Y	N	I	R	E	S	G
Taiwan/2018	MN832850	CPV-2c	.	L	Y	.	G	R	L	A	N	A	I	Y	A	G	Y	N	I	R	E	S	G

Note: The dots (.) represent amino acid residues that are identical to those found in the sequences of FPV-SH2001 and FPV-SH2002.

## Data Availability

Two FPV near full-length sequences from this study were deposited in the Genbank (https://www.ncbi.nlm.nih.gov/genbank/ accessed on 1 March 2023) under accession number of MW650831, MW659466.

## Supplementary Materials

The following supporting information can be downloaded at: https://www.mdpi.com/article/10.3390/cimb45040236/s1, Figure S1: detection and confirmation of FPV isolates through TRFIA test and PCR amplification. (A) Samples were placed into collection tubes and the TRFIA (time resolved fluoro-immunoassay) test strip was used to determine whether FPV was present. Column 1: FPV-SH2001; Column 2: FPV-SH2002; Column 3: Negative control. (B) Samples were also tested using the PCR method. The PCR positive samples presented with a band at 559 bp. Lane M: Trans2K DNA Marker; Lane 1: FPV-SH2001; Lane 2: FPV-SH2002; Lane 3: Negative control. Figure S2: phylogenetic analyses of NS1 amino acid sequences from the isolated FPV strains in this study. The phylogenetic tree was inferred using the Maximum Likelihood method with 1000 bootstrap replicates, using the MEGA-X software (version 10.1.8). Yellow circles designate the FPV sequences identified from the present study; the remaining ones are the reference sequences. Figure S3: phylogenetic analyses of VP2 amino acid sequences from the isolated FPV strains in this study. The phylogenetic tree was inferred using the Maximum Likelihood method with 1000 bootstrap replicates, using the MEGA-X software (version 10.1.8). Red circles designate the FPV sequences identified from the present study; the remaining ones are the reference sequences. Table S1: the results of recombination event detection of VP2 gene. Table S2: the results of recombination event detection of NS1 gene. Table S3: FPV sequence information used in this study.
