# Supplementary material for: Identification and Genome Characterization of Novel Feline Parvovirus Strains Isolated in Shanghai, China
Source: Curr Issues Mol Biol. 2023 Apr 20;45(4):3628–39. doi: 10.3390/cimb45040236 (PMC10136790; doi:10.3390/cimb45040236)
Supplement: Supplementary file 1 [file cimb-45-00236-s001.zip › Supplemental Figures.pdf]

Supplementary Figures

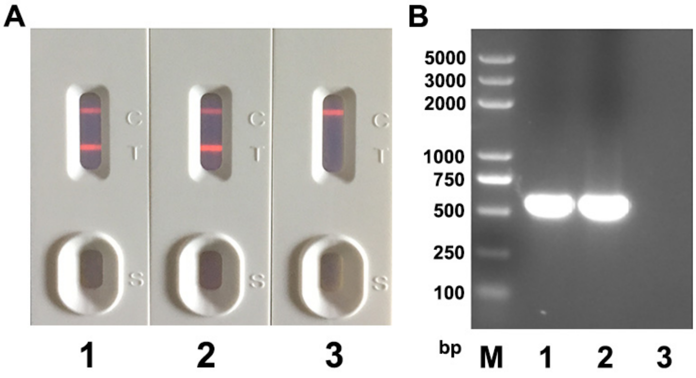

Figure S1. Detection and confirmation of FPV isolates through TRFIA test and PCR amplification. (A) Samples were placed into collection tubes and the TRFIA (time resolved fluoro-immunoassay) test strip was used to determine whether FPV was present. Column 1. FPV-SH2001; Column 2. FPV-SH2002; Column 3. Negative control. (B) Samples were also tested using the PCR method. The PCR positive samples presented with a band at 559 bp. Lane M. Trans2K DNA Marker; Lane 1. FPV-SH2001; Lane 2. FPV-SH2002; Lane 3. Negative control.

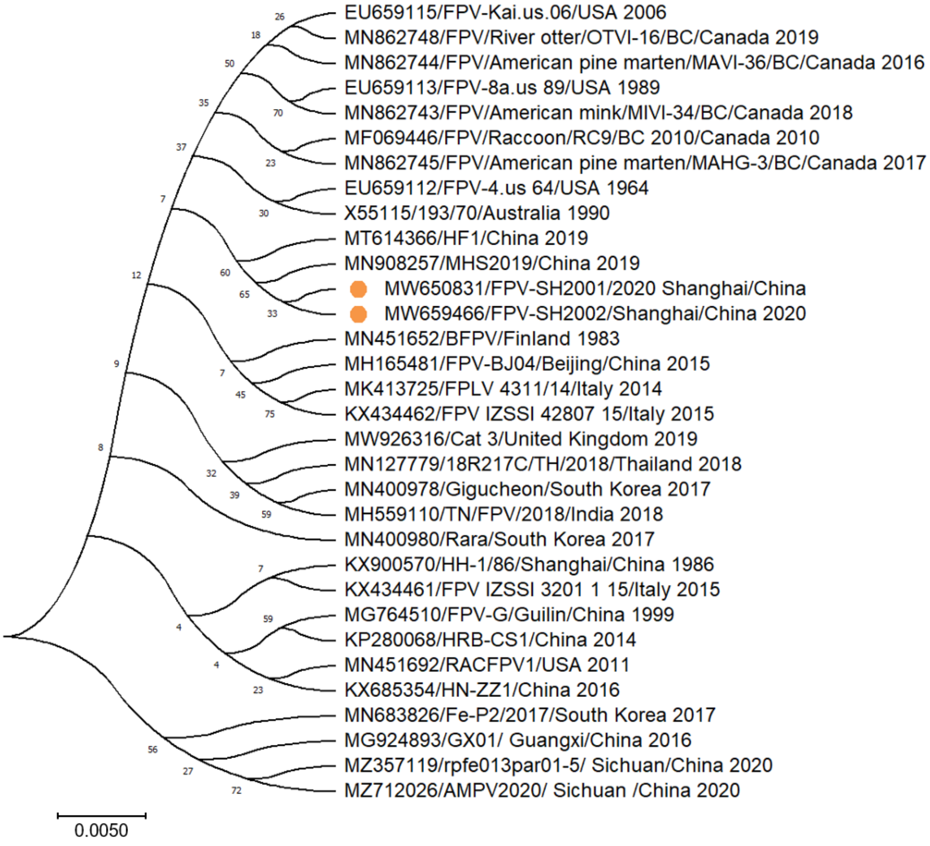

Figure S2. Phylogenetic analyses of NS1 amino acid sequences from the isolated FPV strains in this study. The phylogenetic tree was inferred by using the Maximum Likelihood method with 1,000 bootstrap replicates, using the MEGA-X software (version 10.1.8). Yellow circles designate the FPV sequences identified from the present study, the remain ones are the reference sequences.

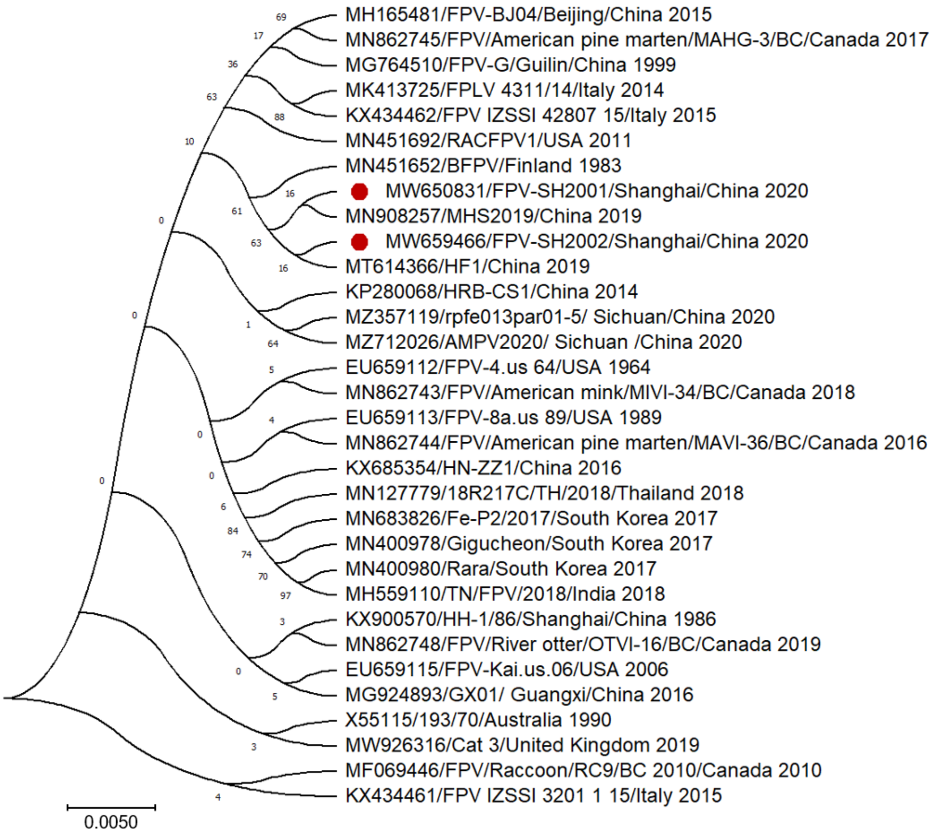

Figure S3. Phylogenetic analyses of VP2 amino acid sequences from the isolated FPV strains in this study. The phylogenetic tree was inferred by using the Maximum Likelihood method with 1,000 bootstrap replicates, using the MEGA-X software (version 10.1.8). Red circles designate the FPV sequences identified from the present study, the remain ones are the reference sequences.
